# Baseline Pro-Inflammatory Cytokine Levels Moderate Psychological Inflexibility in Behavioral Treatment for Chronic Pain

**DOI:** 10.3390/jcm11092285

**Published:** 2022-04-20

**Authors:** Bianka Karshikoff, Jenny Åström, Linda Holmström, Mats Lekander, Mike K. Kemani, Rikard K. Wicksell

**Affiliations:** 1 Department of Social Studies, University of Stavanger, 4021 Stavanger, Norway; 2 Department of Clinical Neuroscience, Karolinska Institutet, 171 77 Stockholm, Sweden; jenny.astrom@ki.se (J.Å.); linda.holmstrom@ki.se (L.H.); mats.lekander@ki.se (M.L.); rikard.wicksell@ki.se (R.K.W.); 3 Medical Unit Medical Psychology, Theme Women’s Health and Allied Health Professionals, Karolinska University Hospital, 171 76 Stockholm, Sweden; mike.kemani@regionstockholm.se; 4 Stress Research Institute, Department of Psychology, Stockholm University, 106 91 Stockholm, Sweden; 5 Pain Clinic, Capio St. Göran Hospital, 112 19 Stockholm, Sweden

**Keywords:** psychological inflexibility, pain interference, cytokine, inflammation, chronic pain, ACT

## Abstract

Background: The medical and scientific communities struggle to understand chronic pain and find effective treatments. Multimodal approaches are encouraging but show significant individual differences. Methods: Seventy-eight persons (56 women) with chronic pain received Acceptance and Commitment Therapy and provided blood samples before and after treatment. The participants completed surveys with the blood sampling. Blood plasma was analyzed for IL-6 and TNF-α levels with the Olink Inflammation Panel (Olink Bioscience Uppsala, Sweden). The treatment effects and moderating effects of low-grade inflammation on changes in outcomes were analyzed using linear mixed models. Results: Pain interference (*p* < 0.001) and psychological inflexibility (*p* < 0.001) improved significantly during treatment, but pain intensity did not (*p* = 0.078). Cytokine levels did not change over the course of the treatment (IL-6/TNF-α *p* = 0.086/0.672). Mean baseline levels of IL-6 and TNF-α moderated improvement in psychological inflexibility during the course of treatment (*p* = 0.044), but cytokine levels did not moderate changes in pain interference (*p* = 0.205) or pain intensity (*p* = 0.536). Conclusions: Higher baseline inflammation levels were related to less improvement in psychological inflexibility. Low-grade inflammation may be one factor underlying the variability in behavioral treatment in chronic pain.

## 1. Introduction

Chronic pain is common, with studies showing prevalence rates of about one in five adults suffering from chronic pain [1]. In addition to a significant impact on emotional well-being and everyday functioning for the many affected, chronic pain is related to substantial societal costs [1,2]. The medical and scientific communities are still struggling to understand the mechanisms underlying the onset and perpetuation of symptoms and find effective treatment strategies. A number of classifications and diagnoses exist, and individual differences in symptom severity and disability are significant even within a specific pain diagnosis [2,3,4], adding to the complexity of the condition. However, some commonalities are seen across chronic pain diagnoses, such as a considerable overlap between chronic pain and psychological suffering [1,2,4]. Increased peripheral and central inflammatory activity has also been demonstrated across a wide range of pain diagnoses [5], such as fibromyalgia [6], neuropathic pain [7] and low back pain [8]. Modern pain treatment acknowledges the complexity of chronic pain disorders and attempts to ease patient suffering with multimodal and individualized approaches. There is a broad consensus on the utility of behavioral interventions to alleviate symptoms and improve health and functioning [9]. However, it is also well known that individuals vary in their response to treatment [9], which calls for a better understanding of the factors influencing treatment outcomes to guide the development of customized interventions with better effects [10].

In the present study, the participants underwent Acceptance and Commitment Therapy (ACT). This exposure-oriented behavioral intervention utilizes acceptance and values-based behavioral strategies to promote the ability to engage in valued activities despite the presence of pain and distress [11,12]. This ability is called psychological (or behavioral) flexibility [13]. Psychological inflexibility, as assessed with, e.g., Psychological Inflexibility in Pain Scale (PIPS) [14], is thus a central treatment target in ACT. Psychological inflexibility is characterized by rigid behavioral repertoires aimed at avoiding pain and related distress [14,15], which may work well in the short term, but over time result in a gradual decrease in functioning without any corresponding alleviation of pain or distress [16]. This type of avoidance was recently shown to predict pain disability and depressive symptoms three years later [17]. ACT does not primarily target pain intensity or related symptoms, but rather the influence of pain on a person’s daily life [11], as assessed by, e.g., The Pain Interference Index (PII) [18]. Previous studies have shown that psychological inflexibility is a more important mediator of changes in pain interference during ACT than, e.g., pain intensity [12]. Thus, by improving the ability to effectively manage pain and related distress (i.e., decrease psychological inflexibility), ACT aims to reduce avoidance strategies and facilitate a broader and more flexible behavioral repertoire in line with personal values and long-term goals (i.e., decrease pain interference). Today, ACT has strong empirical support to improve functioning for persons with chronic pain [19]. However, more studies are needed that investigate for whom, how and under what circumstances these treatment approaches work, i.e., factors that moderate treatment outcome [10]. We propose that ongoing low-grade inflammation may be one such factor.

Experimentally induced inflammation in healthy humans and rodents increases pain sensitivity, decreases mood and changes motivational behaviors, see, e.g., [20,21,22,23] for an overview. Inflammatory activity has been established as a factor in clinically depressed patients [24] and has recently been highlighted as relevant in the perspective of predicting or moderating treatment outcomes in chronic pain [25] and depression [26]. For depression, low-grade inflammatory activity seems to be related to the failure of responding to pharmacological antidepressant treatments specifically [27,28]. Few studies have explored the role of inflammation in behavioral interventions for complex diseases, such as depression and pain. Moreover, only two studies have, to our knowledge, examined the predictive ability of low-grade inflammation on treatment success in chronic pain patients undergoing behavioral interventions. The present study follows a previous trial in which a group of chronic pain patients (n = 41) underwent behavioral treatment (ACT or applied relaxation). Results showed that baseline peripheral low-grade inflammation, operationalized as a combination of interleukin (IL)-6 and tumor necrosis factor (TNF)-α levels in the blood, moderated improvements in pain intensity and psychological inflexibility. Higher baseline inflammatory activity predicted a less favorable treatment outcome [25]. In a recent study in which persons with fibromyalgia participated in treatment comprising mindfulness-based stress reduction (MBSR), a higher ratio of pro- vs. anti-inflammatory cytokines (IL-6/IL-10) was associated with less improvement in psychological inflexibility during treatment [29]. In our study [25], we also saw a small decrease in inflammatory cytokine levels after treatment, which aligns with a meta-analysis on the effects of behavioral therapy on CRP blood levels [30]. In sum, studies evaluating inflammation as a factor influencing the outcome of behavioral treatment for chronic pain are scarce but suggest that inflammation may interfere with improvement. However, results from existing studies are not entirely congruent, and confirmative studies are needed.

Based on our previous study [25], we hypothesized that (1) pain interference and psychological inflexibility would improve following treatment, and pain intensity decrease; (2) levels of low-grade inflammation would decrease during treatment; and (3) baseline inflammatory levels would moderate the effect of treatment on pain interference, pain intensity and psychological inflexibility.

## 2. Materials and Methods

### 2.1. Study Participants and Procedure

Study participants were recruited between 2016–2018 either consecutively via referrals to the Behavioral Pain Medicine Treatment Services at the Karolinska University Hospital or via recruitment advertising. Inclusion criteria comprised the following: ≥18 years of age; pain duration > 6 consecutive months, which did not respond to other treatments and greatly impeded everyday life; stable medication in the last two months; able to communicate in Swedish. Patients were excluded if they participated in a concurrent treatment based on cognitive behavioral therapy (CBT) or presented with severe psychiatric comorbidity that required immediate assessment and/or treatment (e.g., suicidal ideation, psychotic symptoms), or if a spontaneous improvement could be expected. Additional exclusion criteria for phlebotomy were pregnancy, breastfeeding or having given birth within the last year, and hemophilia.

Participants in the face-to-face treatment were assessed using semi-structured interviews at their first visit to the clinic by a pain physician and a psychologist. Patients receiving treatment via the internet were assessed via telephone interview by either a psychologist or candidate psychology student under supervision. When it was unclear if the pain was sufficiently evaluated to participate in the study, a physician was consulted. Psychiatric comorbidity was assessed using the Mini International Neuropsychiatric Interview, version 5 (MINI; Sheehan et al., 1998).

All participants included in the statistical analyses presented in the current paper received ACT. The treatment protocol for face-to-face treatment was delivered weekly during a three-month period [19], and the internet-delivered treatment had learning interactions every weekday for ten weeks [31]. Based on clinical logistics, treatment was made available face-to-face or via the internet. Therapy was provided by psychologists trained in CBT/ACT. When the consent form for study participation was signed, the login information for a first block of questionnaires was sent out. At the commencement of the treatment, the patient was booked for a blood sample, and the second block of questionnaires was activated. At the end of treatment, the second blood sampling was booked with the patient, and the web-based questionnaires were activated. Participants completed the self-reported questionnaires and underwent blood sampling at two time points, i.e., directly prior to treatment onset and directly following the end of treatment. Blood sampling was done in the morning, and questionnaires were filled out in conjunction with blood sampling for most patients (see details below). The study was approved by the Regional Ethical Review Board in Stockholm, Sweden (Permit Number: 2016/1252-31/2), and all participants provided informed consent.

### 2.2. Self-Reportsed Items and Questionnaires

#### 2.2.1. Pain Interference

To assess pain interference, we used the Pain Interference Index (PII), a brief questionnaire that measures the degree to which chronic pain interferes with everyday functioning in central life domains. The PII utilizes a two-week recall period and comprises six items that are rated from 0 (‘Not at all’) to 6 (‘Completely’) using a numerical rating scale, yielding a maximum total score of 36 points. The patient is asked to what degree pain has (1) made it difficult to study/work; (2) made it difficult to do leisure activities; (3) made it difficult to spend time with friends; (4) affected mood; (5) affected the ability to do physical activities; and (6) affected sleep. The psychometric evaluation of the instrument [18] supported a single factor structure and showed satisfactory internal consistency, indicated by a Cronbach’s alpha of 0.85. Furthermore, results from this study also showed that PII explained a significant amount of variance in pain disability, health-related quality of life and depressive symptoms, indicating adequate concurrent criterion validity.

#### 2.2.2. Pain Intensity

Maximum pain intensity in the last two weeks was assessed by a numeric rating scale from 0 (‘No pain’) to 10 (‘Worst imaginable pain’).

#### 2.2.3. Psychological Inflexibility

The Psychological Inflexibility in Pain Scale (PIPS) comprises 12 items, with subscales for ‘Avoidance’ (8 items) and ‘Fusion’ (4 items). Items are rated on a 7-point Likert-scale ranging from 1 (‘Never true’) to 7 (‘Always true’), with higher scores indicating greater psychological inflexibility. In terms of reliability, previous studies have shown Cronbach’s alphas of 0.89 [32] and 0.87 [14], illustrating that the questionnaire has adequate internal consistency. Regarding construct validity, the psychometric evaluations also showed that the PIPS subscales were significantly correlated (*p* < 0.001) with both subscales of the Chronic Pain Acceptance Questionnaire (CPAQ) as well as the activity avoidance subscale of the Tampa Scale of Kinesiophobia (TSK). Finally, PIPS accounted for a significant amount of variance in pain, medication use, work absence, life satisfaction, pain disability, anxiety, depression, kinesiophobia and acceptance [14], indicating adequate concurrent criteria validity.

#### 2.2.4. Anxiety and Depressive Symptoms

To assess symptoms of anxiety and depression, we used the Generalized Anxiety Disorder 7-item scale (GAD-7) and the Patient Health Questionnaire-9 (PHQ-9), respectively. The GAD-7 is a seven-item scale that aims to assess generalized anxiety. Items are scored from 0 (‘Not at all’) to 3 (‘Nearly every day’) and summarized [33]. The PHQ-9 is a nine-item questionnaire that aims to assess the presence of depression and levels of depression over the past two weeks, and questions are scored from 0 to 3 and summarized [34]. 

### 2.3. Cytokine Analyses

Blood was collected in the morning between 8–12 a.m. (non-fasting samples) and processed within two hours. Blood samples were collected just before the treatment period and immediately after the treatment period; max. 50 mL of blood was drawn on both occasions. At elevated CRP values (>10 mg/L), the patients were informed of the results by a physician. Blood plasma was analyzed for IL-6 and TNF-α levels with Proximity Extension Assay (PEA) technology using the Olink Inflammation Panel (Olink Bioscience Uppsala, Sweden) according to the manufacturer’s instructions (for information on all cytokines in the panel, see https://www.olink.com/products/inflammation/ (accessed on 9 March 2022)). Data are expressed as normalized protein expression (NPX), which can be used for multivariate statistical analysis and express relative quantification between samples but are not an absolute quantification. For cytokines, the log-transformed values are used to improve normal distribution. A composite score based on the mean values of IL-6 and TNF-α levels at baseline were used as a measure of ongoing low-grade systemic inflammation, similar to Lasselin et al. [25].

### 2.4. Statistical Analyses

Treatment effects and the moderating effect of low-grade inflammation on changes in outcomes were analyzed using Linear Mixed Models (LMM). In line with the intention to treat, principle maximum likelihood estimation, an approach that uses information from all available observations, was used to estimate parameters and provides unbiased estimates and standard errors in the presence of incomplete data [35], under the assumption that data are missing at random (MAR). Random effects and their associated covariances were retained based on their model contribution, as determined by model comparisons of Akaike’s Information Criterion (AIC; [36]). The assumptions regarding the normal distribution of residuals and homogeneity of variance were assessed, based on visual evaluation of a histogram of model residuals and by a plot of the residuals of the model against the model predicted values, respectively. Moderation, here defined as the effect of a third variable (M) on a relationship between an independent variable (X) and a dependent variable (Y), in such a manner that the relationship between X and Y changes according to the value of M, the moderator variable. In model-building terms, this translated to including an interaction term (X*M), while controlling for M and X in the model.

**Figure 1:**
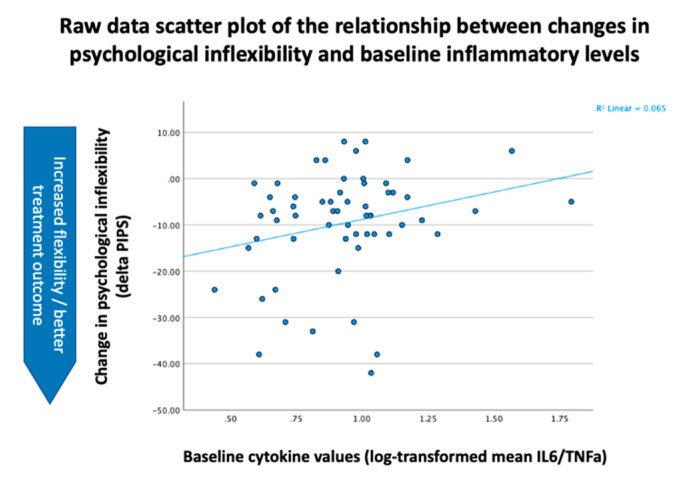
Relationship between changes in psychological inflexibility and cytokine levels at baseline.

Models evaluating treatment effects included age, gender (demographic variables) and Body Mass Index (BMI; clinical background variable) as time-invariant control variables. When evaluating the moderating effect of baseline inflammation (M) on the relationship between time (X) and outcomes (Y), we adjusted for the influence of demographic (gender and age) and clinical background variables (BMI, medication, depression, general anxiety, maximum pain intensity and combined mean levels of IL-6 and TNF-α). Gender, age, BMI and levels of inflammation were included as time-invariant covariates, and maximum pain intensity, depression and general anxiety were included as time-varying covariates. For comparability, control variables were consistent with Lasselin et al. [25]. Lastly, we included the interaction between time (i.e., the treatment effect on the specific outcome variable across assessments) and the combined mean baseline levels of IL-6 and TNF-α. As the analyses were hypothesis-based analyses based on a prior pilot study, we did not correct for multiple comparisons. Descriptive analyses of patient characteristics and self-ratings of the included variables were performed using adequate central values and measures of dispersion. The descriptive statistics in the text and Table 1, Table 2 and Table S1 were analyzed using R version 4.1.2 [37] and the Tableone package [38]. Statistics in Table 3 and Table 4 and Figure 1 were analyzed using IBM SPSS version 26 (IBM Corp. Released 2019. Armonk, NY, USA: IBM Corp).

## 3. Results

### 3.1. Participant Characteristics, Procedure and Missing Data

In total, 78 persons (56 women) with chronic pain were included in the analyses, from a total of 89 included in the study. Of these 78, 56 received internet-based treatment (iACT) and 22 received face-to-face treatment. A total of 50% of the participants who received treatment face-to-face had group sessions. At baseline, participants completed questionnaires and donated blood with a mean of 2.5 days (SD 2.9) before treatment and 1.9 days (SD 2.8) after the end of treatment. One participant was excluded as another treatment was indicated, and one due to extreme values in blood cytokines related to another treatment. Nine participants filled in questionnaires with more than 14 days difference in blood sampling and were therefore excluded from the analysis. Fifty-seven of the 78 included participants completed the treatment, resulting in an attrition rate of 25%. Of these, 52 donated blood both before and after treatment.

Due to logistic difficulties in the clinical setting, PHQ-9 and GAD-7 for the first six patients are missing. Descriptive statistics pertaining to demographic and clinical background variables are presented in Table 1 and Supplementary Table S1, and observed mean values and their respective standard deviations for all included self-reported questionnaires across assessments are presented in Table 2. Visual inspection of the plotted residuals from the model and the plotted model residuals against the model predicted values indicated that assumptions of normality were adequately met for analyses with LMM.

### 3.2. Treatment-Related Effects

As hypothesized, Pain interference (PII) (β = −4.729, *p* < 0.001) and psychological inflexibility (PIPS) (β = −7.166, *p* < 0.001) improved significantly during treatment (Table 3). However, pain intensity (*p* = 0.078) and levels of IL-6 (*p* = 0.086) and TNF-α (*p* = 0.672) did not decrease over time.

### 3.3. Effect of Inflammation on Treatment Outcomes

Inflammatory levels did not moderate changes in pain interference (*p* = 0.205) or pain intensity (*p* = 0.536) (Table 4). Mean levels of pro-inflammatory levels did, however, moderate changes in psychological inflexibility (β = 14.624, *p* = 0.044) during treatment (Table 4). Our results indicate that higher mean baseline levels of IL-6 and TNF-α were related to higher levels of psychological inflexibility during the course of treatment (see Table 4 and Figure 1). This means that patients with lower inflammatory levels showed a greater decrease in psychological inflexibility during ACT treatment as compared to patients with higher inflammatory levels. Sensitivity analyses including only completers yielded a significant relationship between baseline inflammation and changes in PIPS as well, suggesting that this finding is robust.

## 4. Discussion

Several studies have evaluated the inflammatory profile of patients with pain conditions, e.g., [5,7,8,29], and the effects of behavioral treatment on levels of inflammation [30], but few studies have evaluated the effect of inflammation on behavioral treatment for pain [25,29]. Rather than investigating the efficacy of ACT, which has been studied previously by our group [12,13,31,39,40,41,42], the purpose of this study was to understand the potential contribution of baseline low-grade inflammation on the effect of behavioral treatment in patients suffering from chronic pain, assuming ongoing inflammation as one potential variable involved in the large individual variation in treatment efficacy in these patients [9,10].

Results tentatively illustrate that levels of systemic low-grade inflammation moderated the effect of treatment on psychological inflexibility following ACT for chronic pain. These findings are in line with two previous studies, supporting the validity of the findings. Results suggest that baseline inflammation levels can interfere with the effect of treatment on central treatment targets, such as psychological inflexibility. In contrast to our previous study, no moderating effect of inflammation on pain intensity could be seen, nor could we demonstrate a decrease in inflammatory activity due to the treatment. Psychological inflexibility was related to inflammatory levels in the previous study [25], and we investigated pain interference as an outcome variable in this study, as pain interference has been shown to be related to changes in psychological inflexibility in previous research [12]. However, inflammation did not significantly moderate changes in pain interference. The pattern found, of PII and PIPS improving significantly over time during ACT while pain intensity did not, is in line with the previous studies on treatment effects published by our group [12,13,31,39,40,41,42]. Here, the focus is on a biological factor that may affect treatment efficacy.

Our study implies a relationship between inflammatory activity and psychological inflexibility as measured with PIPS. As for the biological mechanisms underlying this finding, we consider the demonstrated effects of inflammation on potential experimental analogues to psychological flexibility. Two behavioral mechanisms that have been discussed in previous research and that may have some bearing on this clinical variable are cognitive/mental flexibility and avoidance behaviors [43]. Cognitive or mental flexibility is often defined as the ability to change behaviors, such as thoughts and actions, in response to situational challenges [43]. It is considered to be an executive function, and may be assessed by, e.g., the Intra/Extra-Dimensional (IED) set shift test [44,45] and the Trail Making Test (TMT) [46,47]. Lasselin et al. showed that in obese individuals, a population often showing ongoing low-grade inflammation, subjects with levels of C-reactive protein (CRP) above 5 mg/L performed worse on the IED test compared to obese subjects with lower levels of CRP or non-obese participants [48]. Furthermore, CRP blood levels have been associated with reduced performance in the TMT in several patient groups experiencing ongoing peripheral inflammatory activity, such as those with peripheral arterial disease [49] and brain cancer [50] and aging populations [51]. Blood cytokine levels also affect motivational aspects in experimental models. Although these interactions are complex [52,53,54], inflammation tends to reduce approach motivation and enhances avoidance motivation [55]. Aversion models in rodents are perhaps the closest pre-clinical approximation to avoidance behaviors [56], although limitations regarding clinical validity need to be kept in mind when interpreting results. Avoidant behavior in relation to inflammation has been studied extensively in rodents, and distinct immune system-to-brain pathways governing this behavior have been identified. For example, Engblom et al. performed a series of inflammation-induced aversion experiments in rodents that identified interferon-gamma [57] and prostaglandin [58] signaling in the endothelium surrounding the blood vessels in the brain as essential for developing aversive behaviors during induced inflammation. Furthermore, stress-induced monocyte recruitment to the brain resulted in anxiety and social avoidance in wild-type mice. In IL-6 knockout mice, avoidance behaviors were prevented despite maintained immune cell recruitment [59]. The concept of avoidance overlaps conceptually with psychological inflexibility [43]. Thus, the present study adds to the growing body of studies exploring the neurobiological mechanisms, pathophysiological consequences and motivational aspects of approach/avoidance behaviors in relation to inflammatory processes. It should be pointed out that the current analyses are based on inflammatory activity, as this follows prior work and hypotheses. Other biological mechanisms that affect behavior, such as the endogenous opioid system, may have effects that go undetected [60,61].

The current study has both strengths and limitations. A major limitation is the lack of a randomized controlled design, which was also connected to clinical logistics. The lack of a control group makes it difficult to assess if the association would be seen in non-treated groups as a spontaneous occurrence over time and/or if this association is present in non-pain cohorts or healthy controls. We can also not draw conclusions about the cytokine levels of this cohort compared to healthy individuals. Furthermore, we cannot assess the effect of the experimental tests such as blood draw and survey completion. Strengths are that the timing and handling of blood samples were sufficiently standardized, and the Olink analysis panel was shown to have sufficient reproducibility for using single sample analyses. However, the study sample is a group of persons with mixed pain diagnoses, and the diagnosis of the primary condition causing pain was not available. This adds some uncertainty to the interpretation of the results, although not undermining the ecological validity, as it represents the majority of chronic pain patients presenting at most pain clinics. There is also variability in the treatment format. Sensitivity analyses show that the type of treatment did not change the main findings. We chose to include both completers and dropouts in the model, as the LMM can account for missing data, and it should be noted that the main finding of this study remains when only completers were analyzed as well, which suggests that the findings are robust. The study adds to the accumulation of knowledge regarding why treatment effects following behavioral interventions differ across individuals. This knowledge may potentially be used to personalize behavioral treatment, e.g., with regard to duration or content of treatment for certain subgroups, such as patients with higher levels of inflammatory mediators.

## 5. Conclusions

Following ACT for chronic pain, low-grade baseline inflammation tentatively moderated the effect of treatment on psychological inflexibility but not pain intensity or pain interference. The results from the present study are consistent with prior research linking ongoing inflammatory activity to psychological inflexibility. More information on inflammatory biomarkers may improve the understanding of the variability in treatment outcome and the ability to identify relevant subgroups of patients who may require adapted treatment approaches. These are important steps toward individualizing interventions and potentially improving the effects of these treatment approaches.

## Figures and Tables

**Table 1:** Demographic and clinical background variables for the included participants.

Variables	Means/Frequencies	SD/Range	N
Age	51.91	14.99	78
BMI	25.40	4.55	78
Pain duration (years)	16.08	13.37	77
Medicine in past two weeks	76 (97.44%)		78

**Table 2:** Means and standard deviations for the included variables at pre- and post-assessment.

Variables	Pre- Treatment		Post-Treatment		N Pre	N Post
	Mean	SD	Mean	SD		
Pain interference (PII)	20.21	8.58	15.27	8.75	78	59
Pain intensity (max pain)	7.50	1.75	6.76	2.28	78	59
Psychological inflexibility (PIPS)	51.36	13.27	42.76	14.48	78	59
Anxiety (GAD-7)	6.10	4.40	4.88	3.63	73	58
Depression (PHQ-9)	9.07	5.24	6.93	4.68	73	58
IL-6	2.57	0.78	2.62	0.74	78	51
TNF-a	2.73	1.17	2.72	1.12	78	51

**Table 3:** Estimates of fixed effects in the model evaluating the treatment-related effect (time) on pain interference, pain intensity, psychological inflexibility and the two measured cytokines.

						95% Confidence Interval	
Dependent Variable	Parameter	β	Std. Error	df	*P*	Lower Bound	Upper Bound
**PII**	Intercept	23.678	3.891	72.452	0.000	15.923	31.433
	Age	−0.106	0.069	70.778	0.128	−0.244	0.031
	Gender	−0.105	2.158	70.755	0.961	−4.409	4.200
	BMI	0.072	0.062	60.629	0.249	−0.052	0.196
	Time	−4.729	1.258	51.268	0.000	−7.253	−2.205
**Pain intensity**	Intercept	6.720	0.829	73.144	0.000	5.068	8.373
	Age	−0.006	0.015	72.466	0.674	−0.036	0.023
	Gender	0.912	0.460	71.332	0.051	−0.005	1.830
	BMI	0.015	0.013	64.723	0.274	−0.012	0.042
	Time	−0.419	0.233	51.486	0.078	−0.887	0.049
**PIPS**	Intercept	63.528	5.711	72.444	0.000	52.144	74.911
	Age	−0.203	0.101	70.687	0.049	−0.405	−0.001
	Gender	−3.623	3.167	70.671	0.257	−9.939	2.693
	BMI	0.005	0.091	60.169	0.952	−0.176	0.187
	Time	−7.166	1.887	51.975	0.000	−10.953	−3.380
**IL-6**	Intercept	1.033	0.140	71.366	0.000	0.754	1.312
	Age	−0.002	0.002	71.491	0.450	−0.007	0.003
	Gender	−0.018	0.078	70.112	0.817	−0.174	0.138
	BMI	−0.002	0.002	66.104	0.422	−0.007	0.003
	Time	0.043	0.030	45.187	0.160	−0.018	0.104
**TNF-α**	Intercept	0.959	0.135	73.237	0.000	0.690	1.229
	Age	−0.001	0.002	73.293	0.646	−0.006	0.004
	Gender	0.061	0.076	72.909	0.426	−0.090	0.212
	BMI	0.000	0.002	71.764	0.974	−0.004	0.005
	Time	0.003	0.015	43.533	0.840	−0.028	0.034

Dependent variables: PII (Pain interference Index); IL-6 (Interleukin 6); TNF-α (Tumor Necrosis Factor alpha); PIPS (Psychological Inflexibility in Pain Scale). Cytokine values are log-transformed.

**Table 4:** Estimates of fixed effects in the model evaluating the moderating effect of low-grade inflammation on pain interference, pain intensity and psychological inflexibility.

						95% Confidence Interval	
Dependent Variable	Parameter	β	Std. Error	df	*p*	Lower Bound	Upper Bound
**PII**	Intercept	−0.379	4.296	80.712	0.930	−8.927	8.169
	Age	−0.029	0.045	67.271	0.520	−0.118	0.060
	Gender	1.252	1.405	71.962	0.376	−1.548	4.052
	BMI	0.012	0.036	50.057	0.733	−0.060	0.085
	Depression ^a^	0.834	0.163	97.585	0.000	0.511	1.157
	Anxiety ^b^	0.281	0.166	94.363	0.094	−0.048	0.611
	Pain intensity	1.056	0.331	97.810	0.002	0.400	1.713
	Medicines	6.996	2.463	71.524	0.006	2.087	11.906
	Inflammation ^c^	−3.315	2.910	95.495	0.257	−9.091	2.462
	Time ^d^	−7.585	3.493	50.008	0.035	−14.602	−0.569
	Inflammation ∗ Time	4.536	3.529	48.656	0.205	−2.556	11.629
**Pain intensity**	Intercept	3.419	1.278	81.999	0.009	0.877	5.961
	Age	−0.002	0.014	69.288	0.896	−0.029	0.026
	Gender	1.413	0.410	65.916	0.001	0.594	2.231
	BMI	0.007	0.011	52.907	0.528	−0.015	0.030
	Depression ^a^	0.167	0.047	96.990	0.001	0.074	0.259
	Anxiety ^b^	−0.035	0.051	94.535	0.490	−0.136	0.066
	Medicines	1.242	0.751	73.503	0.102	−0.253	2.738
	Inflammation ^c^	0.453	0.890	95.329	0.612	−1.314	2.219
	Time ^d^	0.464	1.044	50.772	0.659	−1.632	2.560
	Inflammation ∗ Time	−0.656	1.053	49.290	0.536	−2.772	1.459
**PIPS**	Intercept	35.500	7.646	79.841	0.000	20.284	50.717
	Age	−0.070	0.078	63.655	0.376	−0.226	0.086
	Gender	0.837	2.466	67.533	0.735	−4.085	5.759
	BMI	−0.048	0.061	43.268	0.439	−0.171	0.075
	Depression ^a^	1.141	0.301	97.853	0.000	0.543	1.738
	Anxiety ^b^	0.370	0.301	92.076	0.223	−0.229	0.968
	Pain intensity	−0.545	0.605	92.909	0.370	−1.746	0.657
	Medicines	8.458	4.325	68.297	0.055	−0.172	17.087
	Inflammation ^c^	3.728	5.326	97.158	0.486	−6.843	14.299
	Time ^d^	−19.206	6.990	52.502	0.008	−33.230	−5.183
	Inflammation ∗ Time	14.624	7.080	50.630	0.044	0.409	28.840

Dependent variable: Pain Interference Index (PII); Pain intensity; Psychological Inflexibility in Pain Scale (PIPS). ^a^ Depression (PHQ-9; Patient Health Questionnaire-9); ^b^ Anxiety (GAD-7; Generalized Anxiety Disorder 7-item scale); ^c^ Mean combined baseline values of TNF-α and IL-6, log transformed; ^d^ Corresponds to the treatment effect.

## Data Availability

Data available upon request, please contact the main author.

## Supplementary Materials

The following supporting information can be downloaded at: https://www.mdpi.com/article/10.3390/jcm11092285/s1, Table S1. Additional clinical background variables for all included participants.
