# Supplementary material for: Baseline Pro-Inflammatory Cytokine Levels Moderate Psychological Inflexibility in Behavioral Treatment for Chronic Pain
Source: J Clin Med. 2022 Apr 20;11(9):2285. doi: 10.3390/jcm11092285 (PMC9102370; doi:10.3390/jcm11092285)
Supplement: Supplementary file 1 [file jcm-11-02285-s001.zip › jcm-1654722-supplementary.pdf]

Supplementary Table S1. Additional clinical background variables for all included participants.

Characteristics	N (%)/ Mean (SD)	N
Pain localizationsϕ		
Headache	38 (48.7%)	78
Facial	8 (10.3%)	78
Teeth jaw	19 (24.4%)	78
Neck	47 (60.3%)	78
Back	55 (70.5%)	78
Chest	10 (12.8%)	78
Abdominal	26 (33.3%)	78
Genital	5 (6.4%)	78
Arm	35 (44.9%)	78
Hand	29 (37.2%)	78
Leg	41 (52.6%)	78
Foot	36 (46.2%)	78
Other pain	31 (39.7%)	78
Entire body	26 (33.3%)	78
Moving pain	44 (56.4%)	78
Amount of localizations		
One localization	7 (9.0%)	78
Multiple localizations	71 (91.0%)	78
Pain medications ψ		
Opioids	23 (29.5%)	78
Antiepileptics	17 (21.8%)	78
NSAID	21 (26.9%)	78
Anti-depressants	23 (29.5%)	78
Sedatives/relaxants	18 (23.1%)	78
Specific migraine medications	5 (6.4%)	78
Other analgesics	36 (46.2%)	78
No medication past two weeks	2 (2.6%)	78

ϕ Participants could report more than 1 localization.

ψ Participants could report more than 1 medication.
